# Manipulation of components of the renin angiotensin system in renal proximal tubules fails to alter atherosclerosis in hypercholesterolemic mice

**DOI:** 10.3389/fcvm.2023.1250234

**Published:** 2023-08-16

**Authors:** Masayoshi Kukida, Naofumi Amioka, Dien Ye, Hui Chen, Jessica J. Moorleghen, Ching-Ling Liang, Deborah A. Howatt, Yuriko Katsumata, Motoko Yanagita, Hisashi Sawada, Alan Daugherty, Hong S. Lu

**Affiliations:** ^1^ Saha Cardiovascular Research Center, University of Kentucky, Lexington, KY, United States; ^2^ Sanders-Brown Center on Aging, University of Kentucky, Lexington, KY, United States; ^3^ Department of Biostatistics, University of Kentucky, Lexington, KY, United States; ^4^ Department of Nephrology, Kyoto University Graduate School of Medicine, Kyoto, Japan; ^5^ Institute for the Advanced Study of Human Biology (WPI-ASHBi), Kyoto University, Kyoto, Japan; ^6^ Saha Aortic Center, University of Kentucky, Lexington, KY, United States; ^7^ Department of Physiology, University of Kentucky, Lexington, KY, United States

**Keywords:** angiotensin, angiotensinogen, renin, angiotensin-converting enzyme, AT1 receptor, atherosclerosis, kidney, proximal tubules

## Abstract

**Background and objective:**

Whole body manipulation of the renin-angiotensin system (RAS) consistently exerts profound effects on experimental atherosclerosis development. A deficit in the literature has been a lack of attention to the effects of sex. Also, based on data with gene-deleted mice, the site of RAS activity that influences lesion formation is at an unknown distant location. Since angiotensin (AngII) concentrations are high in kidney and the major components of the RAS are present in renal proximal tubule cells (PTCs), this study evaluated the role of the RAS in PTCs in atherosclerosis development.

**Methods and results:**

Mice with an LDL receptor −/− background were fed Western diet to induce hypercholesterolemia and atherosclerosis. We first demonstrated the role of AT1 receptor antagonism on atherosclerosis in both sexes. Losartan, an AngII type 1 (AT1) receptor blocker, had greater blood pressure-lowering effects in females than males, but equivalent effects between sexes in reducing atherosclerotic lesion size. To determine the roles of renal AT1a receptor and angiotensin-converting enzyme (ACE), either component was deleted in PTCs after weaning using a tamoxifen-inducible Cre expressed under the control of an *Ndrg1* promoter. Despite profound deletion of AT1a receptor or ACE in PTCs, the absence of either protein did not influence development of atherosclerosis in either sex. Conversely, mice expressing human angiotensinogen and renin in PTCs or expressing human angiotensinogen in liver but human renin in PTCs did not change atherosclerotic lesion size in male mice.

**Conclusion:**

Whole-body AT1R inhibition reduced atherosclerosis equivalently in both male and female mice; however, PTC-specific manipulation of the RAS components had no effects on hypercholesterolemia-induced atherosclerosis.

## Introduction

Angiotensin II (AngII) is the principal bioactive peptide of the renin-angiotensin system (RAS), which is produced from angiotensinogen (AGT) through sequential cleavage by renin and angiotensin-converting enzyme (ACE). AngII is a potent contributor to atherosclerosis through binding to AngII type 1 receptor (AT1R in humans and AT1aR in mice). Sex differences in atherosclerosis have been noted in previous studies (1). However, most mouse studies focus on effects of the RAS components on atherosclerosis in male mice, while there is a paucity of studies that performed simultaneous comparisons of the efficacy of RAS inhibition on atherosclerosis in both male and female mice.

Whole body manipulation of the RAS has generated a highly consistent literature on its role in regulating experimental atherosclerosis (2, 3). However, there has not been a clearly defined location for the production of AngII that influences atherosclerosis or the cellular location for activation of AT1aR to promote the disease. The RAS components are present throughout the body. Classically, the principal location for producing AngII has been liver for AGT, renal juxtaglomerular cells for renin, and lung for ACE (4). Of note, many tissues and cell types have AT1aR and every component to generate AngII. These RAS components are also present in atherosclerotic lesions and resident cell types of the vascular wall (5–9). Although one prevailing hypothesis has been that the RAS in circulating leukocytes and/or resident cell types of the vessel wall exerts an important role in atherosclerosis (5–9), deletion of AT1aR on macrophages (a major cell type in atherosclerosis) or resident cell types of the arterial wall does not affect atherosclerosis (9–11), indicating that AngII-AT1aR stimulation may not act directly on the vascular wall to promote atherosclerosis.

Our studies have demonstrated that either whole-body or liver-specific inhibition of AGT synthesis decreases atherosclerosis and reduces renal, but not plasma, AngII concentrations (12–14). Further, inhibition of megalin, an endocytic receptor for many molecules including AGT and renin on renal proximal tubules, results in profound reductions of renal AngII concentrations and atherosclerosis (12). Although these findings implicate that the renal RAS contributes to atherosclerosis, there has no direct evidence to support this hypothesis. Since AT1aR and ACE are abundant in renal proximal tubule cells (PTCs), the present study used an inducible PTC-specific genetic mouse strain to delete either AT1aR or ACE in PTCs and determined whether deleting either component affects hypercholesterolemia-induced atherosclerosis in LDL receptor −/− mice. In addition to genetic deletion of AT1aR or ACE, we manipulated AGT and renin by implementing human AGT and renin transgenes in PTCs (15) or human AGT in liver with human renin in PTCs of LDL receptor −/− mice.

## Materials and methods

Detailed Materials and Methods are available in the manuscript or the online-only Data Supplement. The data that support the findings reported in this manuscript are available in the Supplementary Excel File.

### Animals

LDL receptor −/− (Stock # 002207), ROSA26R^LacZ^ (Stock # 003474), and ROSA26R^mT/mG^ (Stock # 007676) mice were purchased from The Jackson Laboratory (Bar Harbor, ME, USA). *Agtr1a* (Angiotensin II type 1a receptor) floxed (*Agtr1a* f/f) mice (10, 16) and *Ace* (angiotensin-converting enzyme) floxed (*Ace* f/f) mice (17, 18) were developed by the authors and maintained for in house breeding. *Ndrg1*-CreERT2 breeding pairs were provided by Dr. Motoko Yanagita at Kyoto University in Japan (19). Transgenic mice expressing human AGT or human renin in PTCs driven by a kidney androgen-related protein promoter (*Kap*-hAGT and *Kap*-hREN, respectively) were provided by Dr. Curt Sigmund (15, 20).

The breeding strategy for generating PTC-specific AT1aR or ACE deficient mice are shown in Table 1.

**Table 1:** Breeding strategy for *Agtr1a* f/f or *Ace* f/f × *Ndrg1*-CreERT2 mice.

	Female parent	Male parent	Offspring for breeding or experiments
F0	f/f	*Ndrg1*-CreERT2^+/0^	Female f/+ × *Ndrg1*-CreERT2^0/0^ Male f/+ × *Ndrg1*-CreERT2^+/0^
F1	f/+ × *Ndrg1*-CreERT2^0/0^	f/+ × *Ndrg1*-CreERT2^+/0^	Female f/f × *Ndrg1*-CreERT2^0/0^ Male f/f × *Ndrg1*-CreERT2^+/0^
F2	f/f × *Ndrg1*-CreERT2^0/0^	f/f × *Ndrg1*-CreERT2^+/0^	f/f × *Ndrg1*-CreERT2^0/0^ f/f × *Ndrg1*-CreERT2^+/0^ Both males and females—Experiment

To validate *Ndrg1*-Cre specificity, male *Ndrg1*-CreERT2 mice were bred with either female ROSA26R^LacZ^ or ROSA26R^mT/mG^ mice to generate ROSA26R^LacZ^ × *Ndrg1-*CreERT2 or mT/mG × *Ndrg1-*CreERT2 mice. To induce Cre activity, mice at 4–6 weeks of age were injected intraperitoneally with tamoxifen (150 mg/kg/day) for five consecutive days. Genotypes were determined prior to weaning and verified after termination by PCR of Cre transgene.

All mice were maintained in a barrier facility on a light:dark cycle of 14:10 h and fed a normal mouse laboratory diet after weaning. All study mice for atherosclerosis were in an LDL receptor −/− background. For mice containing *Ndrg1-*CreERT2 transgene, to induce Cre activity, mice at 4–6 weeks of age were injected intraperitoneally with tamoxifen 150 mg/kg/day for 5 days. Two weeks after the last injection of tamoxifen, mice were fed a diet containing saturated fat (milk fat 21% wt/wt) and cholesterol (0.2% wt/wt; Diet # TD.88137, Envigo, termed “Western diet”). Since we hypothesized that PTC-specific deletion of AT1aR or ACE would reduce atherosclerosis, we fed these mice Western diet for 12 weeks. This feeding duration leads to profound atherosclerosis in the ascending and arch aortic region as reported previously (11, 21–23).

To activate human AGT and/or renin transgenes in PTCs, testosterone pellets (20 mg/pellet for 90-day release or 15 mg/pellet for 60-day release) were implanted subcutaneously. Two weeks later, all mice were fed Western diet for either 8 or 6 weeks. LDL receptor −/− mice fed Western diet for 6–8 weeks have early to intermediate stages of atherosclerosis. We hypothesized that increased human AGT and renin in PTCs leads to augmentation of atherosclerosis. Therefore, a shorter duration of Western diet feeding (either 6 or 8 weeks) was used.

To induce synthesis of human AGT in hepatocytes, an adeno-associated viral vector (AAV; 3 × 10^10^ genome copies/mouse) serotype 2/8 containing human AGT with a liver-specific promoter thyroxine-binding globulin (TBG) was injected intraperitoneally. Three groups of male littermates were administered testosterone to activate human renin expression in PTCs: (1) wild type mice administered null.AAVs, (2) *Kap-*hREN transgenic mice administered null.AAVs, and (3) *Kap*-hREN transgenic mice administered AAVs containing human AGT.

Both male and female mice were used for the experiments reported in this manuscript following the recent ATVB Council Statement (23) except for the data reported in Figures 5, 6. Testosterone administration is required to activate human AGT or renin transgene. Shortly after testosterone pellet implantation, many female mice suffered from uterine prolapse that required euthanasia prior to the endpoint. Therefore, we excluded female mice for studies presented in Figures 5, 6. Euthanasia before the endpoint was performed using CO_2_ (fill rate >50% of the chamber volume/minute) followed by cervical dislocation.

At termination, mice were euthanized by a ketamine (90 mg/kg) and xylazine (10 mg/kg) cocktail. All animal studies performed were approved by the University of Kentucky Institutional Animal Care and Use Committee (Protocol number 2018–2968).

### Administration of losartan

Mini osmotic minipumps (Alzet Model 1004, Durect Corporation) filled with either water (vehicle) or losartan (12.5 mg/kg/day, Cat # 61188, MilliporeSigma) were implanted subcutaneously into LDL receptor −/− mice, as described in our previous studies (24). After anesthetized using inhaled isoflurane (2%–3% vol/vol), mice at ∼9–10 weeks of age were implanted with mini osmotic pumps. Pumps were replaced after 4 and 8 weeks of the first pump implantation. All mice were fed Western diet for 12 weeks starting one day after the first pump implantation.

### Plasma profiles

Blood samples were collected with EDTA (final concentration: ∼1.8 mg/ml) by submandibular bleeding during experiments and right ventricular punctures at termination. Then, plasma was collected by centrifugation at 3,000 rcf for 10 min, 4°C, and stored at −80°C. Plasma renin concentrations were evaluated by quantifying AngI generation after incubation with exogenous recombinant mouse AGT in the presence of EDTA (Cat # 51201, Lonza, final concentration: ∼14 mM) and PMSF for 60 min at 37°C. The reaction was terminated by placing samples at 100°C for 5 min and generated AngI was measured using a plasma renin activity ELISA kit (Cat # IB59131, IBL America). Plasma total cholesterol concentrations were measured using a commercial enzymatic kit (Cat # 999–02601, Wako Chemicals USA or Cat # C7510–120, Pointe Scientific).

### Blood pressure measurement

Systolic blood pressure was measured on conscious mice by a non-invasive tail-cuff system (BP-2000, Visitech) following our standard protocol (25). Data were collected at the same time each day for three consecutive days. Criteria for accepted data were systolic blood pressure between 70 and 200 mmHg and standard deviation <30 mmHg for at least 5 successful recorded data/mouse/day. The mean systolic blood pressure of each mouse from the 3-day measurements was used for data analysis.

### Quantification of atherosclerosis

Atherosclerotic lesions were evaluated on the intimal surface area of the aorta with an *en face* method following the AHA Statement (23) and our standard protocol (22). Briefly, the aorta was dissected and fixed with neutrally buffered formalin (10% wt/vol) overnight at room temperature. Periaortic tissues were removed gently. Then the intimal surface was exposed by a longitudinal cut, and pinned on a black wax surface. Images of *en face* aortas were taken using a Nikon digital camera (Nikon digital sight DS-Ri1) with a mm ruler for calibration. Lesions were traced manually from the beginning of the ascending region to the descending aortic region that is 1 mm distal from the left subclavian artery using Nikon NIS-Elements software (NIS-Elements AR 5.11.00.) under a dissecting microscope. Lesion size was calculated as percent lesion area using the following formula.

$$\mathrm{Percent}\;\mathrm{lesion}\;\mathrm{area}=\frac{\mathrm{Atherosclerotic}\;\mathrm{lesion}\;\mathrm{area}\;(mm^{2})}{\mathrm{Intimal}\;\mathrm{area}\;\mathrm{of}\;\mathrm{the}\;\mathrm{aorta}\;(mm^{2})}\times 100$$

### Cryosectioning and imaging of mouse kidneys

Isolated whole kidneys from ROSA26R^mT/mG ^× *Ndrg1*-CreERT2 mice were placed immediately in paraformaldehyde (PFA, 4% wt/vol) solution and fixed overnight. Following fixation, kidneys were incubated with 30% sucrose at 4°C overnight and embedded in O.C.T. for cryosectioning. Tissues were cut (10 µm/section) on a Leica CM1860 cryostat (Leica Biosystems). After washing with PBS, sections were mounted with mounting media containing DAPI, and images were captured using Axioscan 1 or 7 (Zeiss) or a confocal microscope (A1 HD25/A1R HD25, Nikon).

### RNA isolation and quantitative PCR

Total RNA of the renal cortex was extracted using a commercial kit (Cat # AS1340, Promega) configured with a Maxwell RSC Instrument (Promega). To quantify mRNA abundance, total RNA was reversely transcribed with an iScript™ cDNA Synthesis kit (Cat # 170–8891, Bio-Rad), and quantitative PCR (qPCR) was performed using a SsoFast™ EvaGreen® Supermix kit (Cat # 172–5204, Bio-Rad) on a Bio-Rad CFX96 real-time system. Data were analyzed using *ΔΔ*Ct method and normalized by the geometric mean of 3 reference genes [*Actb*, *Gapdh*, and *Rplp2* (or *Ppia*)].

### In situ hybridization

The distribution of mouse *Agtr1a* and human renin (*hREN*) mRNA in kidney tissue sections was examined by RNAscope® following the manufacturer's instructions (Advanced Cell Diagnostics). After fixation using 4% PFA, kidney tissues were processed and embedded into paraffin, and cut at a thickness of 4 µm. Subsequently, sections were deparaffinized using xylene followed by 2 washes with absolute ethanol. Target retrieval (Cat # 26043–05) was performed for 30 min at 100°C, and followed by a protease (Cat # 322331) incubation step for 15 min at 40°C. Target mRNA was hybridized with mouse *Agtr1a* (Cat # 481161) or human REN probe (Cat # 401921) for 2 h at 40°C, and amplified signals were detected using diaminobenzidine (Cat # 322310). Hematoxylin was used to stain nuclei. Images were captured using a Nikon Eclipse Ni microscope or Zeiss Axioscan 1 or 7.

### Immunostaining

Immunostaining was performed on paraffin-embedded sections (4 µm) to determine the distribution of mouse ACE and human AGT in the kidney. Deparaffinized sections were incubated with an antigen retrieval reagent (Cat # HK547-XAK; BioGenex) for 20 min at 90°C. Non-specific binding sites were blocked using goat serum for 20 min at room temperature. Sections were then incubated with rabbit anti-mouse ACE antibody (Cat # ab254222; abcam) or anti-human AGT antibody (Cat # ab276132; abcam) diluted in primary antibody diluent (Cat #: GTX28208; GeneTex) for 12 h at 4°C. Goat anti-rabbit IgG conjugated with horseradish peroxidase (Cat # MP-7451; Vector laboratories) was used as the secondary antibody. ImmPACT® AEC (Cat # SK4205; Vector) was used as the chromogen, and hematoxylin (Cat # 26043–05; Electron Microscopy Sciences) was used for counterstaining. Histological images were captured using either an Eclipse Ni microscope or Zeiss Axioscan 1 or 7.

### Statistical analysis

All statistical analyses were performed using SigmaPlot 14.5 or 15 or R Statistical Software (v4.1.1; R Core Team 2021). The assumption of normality was examined using QQ-plot and Shapiro–Wilk test. The homogeneous group variance assumption was assessed by Bartlett test. In studies including both sexes, two-way ANOVA was used to evaluate the interaction between sex and treatment or genotypes, and compare mean difference between groups in each sex, and *P*-value was adjusted using the Bonferroni method in the *post hoc* test. Non-normal distributed data were log-transformed to meet the normal distribution assumption. When heteroscedasticity was present, White-corrected covariance matrix was incorporated into the two-way ANOVA. In studies using only male mice with N ≤5/group, Mann–Whitney *U* test and Kruskal–Wallis one-way ANOVA on Ranks test were used in comparisons between two groups and among three- or four-groups, respectively. For N >5/group, mean comparisons were performed by one-way ANOVA test or Kruskal–Wallis one-way ANOVA on Ranks test depending on data distribution. *P* < 0.05 was considered statistically significant.

## Results

### Losartan reduced atherosclerosis in male and female hypercholesterolemic mice

Losartan inhibits both AT1aR and AT1bR, although our previous study demonstrated that AT1aR, but not AT1bR, contributes to atherosclerosis in mice (26). Losartan reduced atherosclerotic lesions in a dose-dependent manner (21). Losartan (12.5 mg/kg/day) led to a modest reduction of systolic blood pressure and atherosclerosis in male hypercholesterolemic mice, as demonstrated previously (21). To examine whether losartan had equivalent responses between sexes, male and female LDL receptor −/− mice were fed Western diet and infused with either water (Vehicle) or losartan (12.5 mg/kg/day) for 12 weeks.

**Figure 1:**
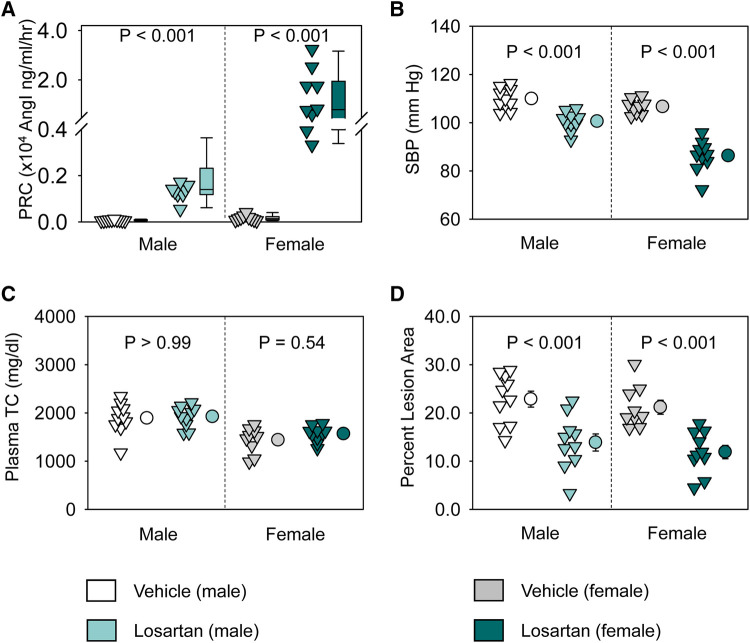
Losartan reduced systolic blood pressure and attenuated atherosclerosis in both male and female LDL receptor −/− mice. Both male and female LDL receptor −/− mice were fed Western diet and infused with either vehicle or losartan (12.5 mg/kg/day) for 12 weeks. *N* = 9–10/group. (**A**) Plasma renin concentrations (PRC), (**B**) systolic blood pressure (SBP), (**C**) plasma total cholesterol concentrations (TC), and (**D**) percent atherosclerotic lesion area. Data of PRC was log-transformed to meet normal distribution assumptions for statistical analysis. Two-way ANOVA was used to evaluate the interaction between sex and treatment. *P*-value was adjusted using the Bonferroni method in the *post hoc* test that examined the effect of losartan on each parameter in both sexes. *P* (sex × treatment) = 0.008 for (**A**) and 0.001 for (**B**), respectively.

Losartan significantly increased plasma renin concentrations and decreased systolic blood pressure in both sexes (Figures 1A,B). Of note, losartan led to more profound increases of plasma renin concentrations and greater reduction of systolic blood pressure in female than in male mice (Figures 1A,B). Losartan did not change plasma total cholesterol concentrations in either male or female mice (Figure 1C), but attenuated atherosclerotic lesion size equivalently in male and female mice (Figure 1D). This study provides direct evidence that AT1R blockade has comparable effects on atherosclerosis in both sexes.

### Validation of PTC-specific deletion induced by *Ndrg1*-CreERT2 activation

**Figure 2:**
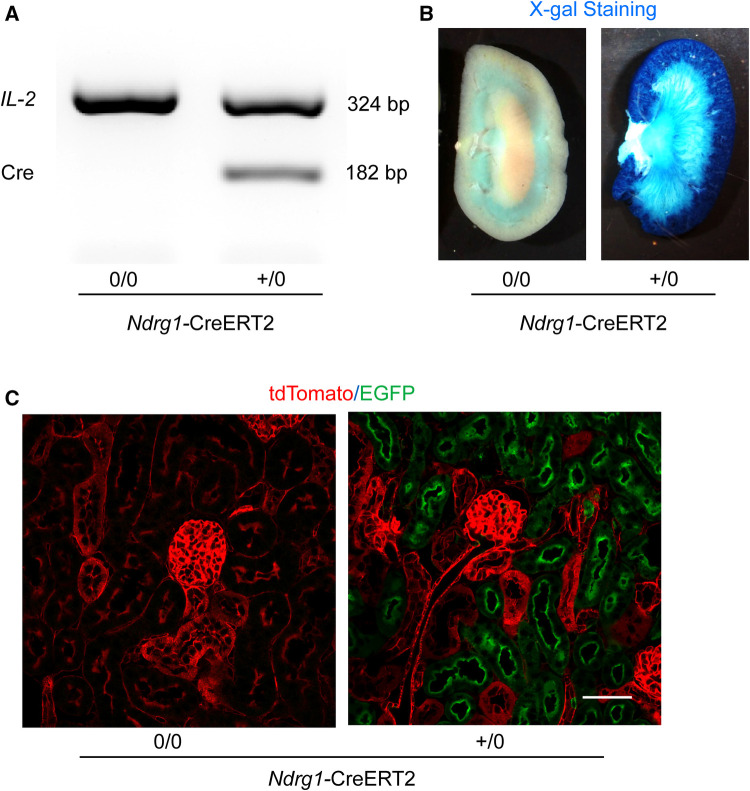
Validation of *Ndrg1*-Cre activation and distribution. *Ndrg1-*CreERT2^0/0^ and *Ndrg1-*CreERT2^+/0^ mice were injected intraperitoneally with tamoxifen 150 mg/kg/day for 5 consecutive days. Kidneys were harvested two weeks after the completion of tamoxifen injection. (**A**) PCR for detecting Cre recombinase in tail DNA, (**B**) X-gal staining of mouse kidneys, and (**C**) membrane-localized tdTomato and EGFP proteins detected by confocal microscopy in kidney sections.

Genotyping of Cre was determined for all study mice (Figure 2A). To determine the specificity of *Ndrg1*-driven Cre, *Ndrg1*-CreERT2^+/0^ mice were bred with either ROSA26R^LacZ^ or ROSA26R^mT/mG^ reporter mice. These mice at 4–6 weeks of age were injected with 150 mg/kg/day of tamoxifen for 5 consecutive days. Kidneys were harvested at 2 weeks after completion of tamoxifen injection. X-gal staining revealed the presence of β-galactosidase activity predominantly in renal cortex of ROSA26R^LacZ ^× *Ndrg1*-CreERT2^+/0^ mice (Figure 2B). In kidneys from ROSA26R^mT/mG ^× *Ndrg1*-CreERT2^+/0^ mice, fluorescent images were captured using a confocal microscope. tdTomato was exclusively expressed in all renal cells in ROSA26R^mT/mG ^× *Ndrg1*-CreERT2^0/0^ mice, while Cre activation in ROSA26R^mT/mG ^× *Ndrg1*-CreERT2^+/0^ mice induced EGFP expression on proximal tubules, including proximal convoluted tubules and some proximal straight tubules. These results confirm that *Ndrg1*-driven Cre is predominantly expressed in proximal convoluted tubules (S1 and S2) of *Ndrg1*-CreERT2^+/0^ mice (Figure 2C), consistent with what was reported by Endo and colleagues (19).

### AT1aR deletion in renal PTCs had no effect on atherosclerosis in both male and female mice

**Figure 3:**
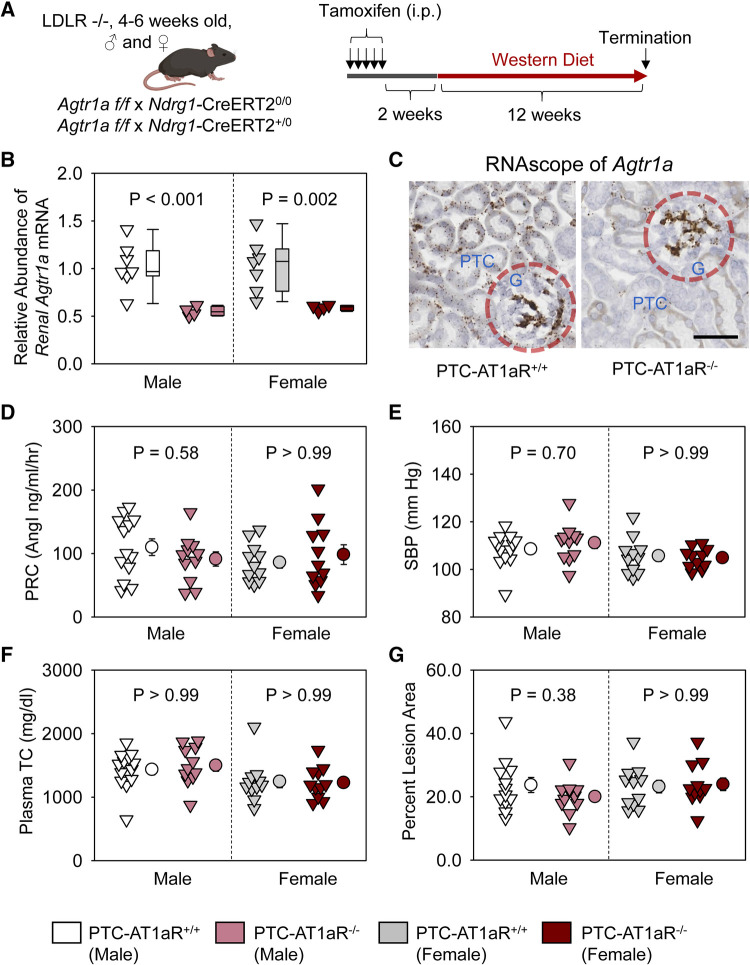
AT1aR deletion in PTCs had no effect on atherosclerosis in hypercholesterolemic mice. (**A**) Experimental protocol, (**B**) qPCR of renal *Agtr1a*, (**C**) RNAscope of renal *Agtr1a*, (**D**) plasma renin concentrations (PRC), (**E**) systolic blood pressure (SBP), (**F**) plasma total cholesterol concentrations (TC), and (**G**) percent atherosclerotic lesion area. *N* = 11–13/group. Two-way ANOVA was used to evaluate the interaction between sex and treatment (**B,D–G**). *P*-value was adjusted using the Bonferroni method in the *post hoc* test that examined the effect of AT1aR deletion in PTCs. G, glomerulus; PTC, proximal tubule cells.

To examine whether AT1aR in PTCs contributes to atherosclerosis, PTC-AT1aR^−/−^ mice and their wild type littermates in an LDL receptor −/− background were fed Western diet for 12 weeks beginning 2 weeks after completion of tamoxifen injection (Figure 3A). Renal mRNA abundance of *Agtr1a* in PTC-AT1aR^−/−^ mice was approximately 45% of that in their PCT-AT1aR^+/+^ littermates (Figure 3B). Unfortunately, no antibodies have been demonstrated to authentic staining of AT1aR protein in mice (27–30). As an alternative mode of verifying PTC-specific AT1aR deletion in PTC-AT1aR^−/−^ mice after Cre activation, we performed RNAscope using paraffin-embedded renal sections. AT1aR mRNAs were detected in PTCs, glomeruli, and interstitial cells of PTC-AT1aR^+/+^ mice (Figure 3C), consistent with previous reports (31). While mRNA of *Agtr1a* in glomeruli and interstitial cells was observed, its mRNA in PTCs was not detected in PTC-AT1aR^−/−^ mice (Figure 3C). These results confirm that *Ndrg1*-Cre activation led to effective AT1aR deletion in PTCs of PTC-AT1aR^−/−^ mice.

Plasma renin concentrations (Figure 3D) and systolic blood pressure (Figure 3E) were not different between PCT-AT1aR^+/+^ and PCT-AT1aR^−/−^ littermates within each sex. Western diet induced hypercholesterolemia in both sexes, but plasma total cholesterol concentrations were not different (Figure 3F) and percent atherosclerotic lesion area was equivalent between the two genotypes within each sex (Figure 3G).

### Renal PTC-specific deletion of ACE did not affect atherosclerosis in both male and female mice

**Figure 4:**
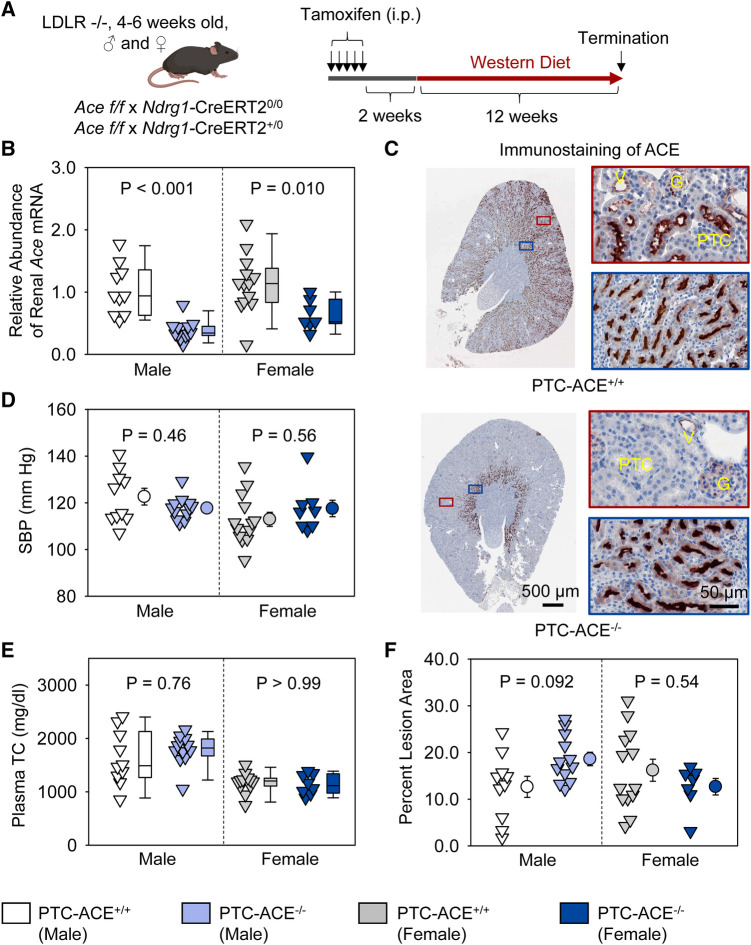
ACE deletion in PTCs had no effect on atherosclerosis in hypercholesterolemic mice. (**A**) Experimental protocol, (**B**) qPCR of renal *Ace*, (**C**) immunostaining of ACE in kidney, (**D**) systolic blood pressure (SBP), (**E**) plasma total cholesterol concentrations (TC), and (**F**) percent atherosclerotic lesion area. *N* = 7–13/group. Two-way ANOVA was used to evaluate the interaction between sex and genotypes (**B,D–F**). White-corrected covariance matrix was incorporated into the two-way ANOVA for analyzing data with heteroscedasticity (**B,E**). G, glomerulus; PTC, proximal tubule cells; V, vessel.

To determine whether ACE in PTCs contributed to atherosclerosis, PTC-ACE^−/−^ mice and their PTC-ACE^+/+^ littermates in an LDL receptor −/− background were fed Western diet for 12 weeks beginning at 2 weeks after completion of tamoxifen injection (Figure 4A). Renal mRNA abundance of ACE was 60% lower in male and 40% lower in female PTC-ACE^−/−^ mice, compared to their sex- and age-matched PTC-ACE^+/+^ littermates, respectively (Figure 4B). Deletion of ACE protein in PTCs was also confirmed by immunostaining of ACE in kidney (Figure 4C). PTC-specific ACE deletion did not change systolic blood pressure in either male or female mice (Figure 4D). Western diet induced hypercholesterolemia in both male and female mice. Plasma total cholesterol concentrations were comparable between the two genotypes in either sex (Figure 4E). Despite the profound deletion of ACE in PTCs, atherosclerotic lesion size was not reduced in either sex (Figure 4F).

### Presence of human AGT and human renin in renal PTCs had no effect on atherosclerosis in male mice

**Figure 5:**
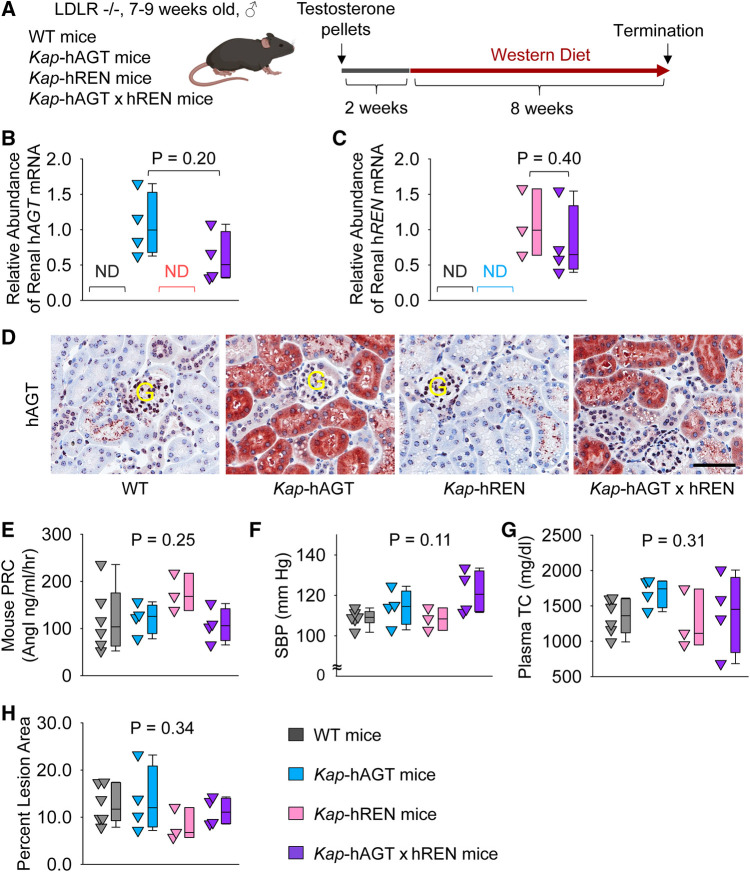
Human AGT and renin in PTCs had no effect on atherosclerosis in hypercholesterolemic mice. (**A**) Experimental protocol, (**B**) qPCR of renal human AGT (h*AGT)*, (**C**) qPCR of renal human renin (h*REN*), (**D**) immunostaining of human AGT (hAGT) in kidney, (**E**) plasma renin concentrations (PRC), (**F**) systolic blood pressure (SBP), (**G**) plasma total cholesterol concentrations (TC), and (**H**) percent atherosclerotic lesion area. *N* = 3–6/group. Mann–Whitney *U* test was performed to determine the difference in the abundance of renal h*AGT* (**B**) or h*REN* (**C**) mRNA between two groups. Kruskal–Wallis one-way ANOVA on Ranks test was performed to determine the difference in each parameter among four groups (**E–H**). ND, not detectable; G, glomerulus.

To activate human AGT and/or renin transgene in PTCs, testosterone pellets were implanted subcutaneously (Figure 5A). To examine whether co-presence of human AGT and renin in PTCs contributed to atherosclerosis, *Kap-*hAGT, *Kap-*hREN, *Kap-*hAGT × *Kap*-hREN*,* and their wild type littermates in an LDL receptor −/− background were fed Western diet for 8 weeks beginning two weeks after testosterone pellet implantation. As confirmed by qPCR of human AGT in mouse kidney (Figure 5B), mRNA abundance of human AGT was high in mice with *Kap-*hAGT or *Kap-*hAGT × *Kap*-hREN, but not detectable in mice with *Kap-*hREN or their wild type littermates. mRNA abundance of human renin (Figure 5C) was high in mice with *Kap*-hREN or *Kap-*hAGT × *Kap*-hREN, but not detectable in mice with *Kap*-hAGT or their wild type littermates. Presence of human AGT protein was also confirmed by immunostaining of human AGT that does not have cross-reaction with mouse AGT (Figure 5D). Unfortunately, we were not able to demonstrate the presence of human renin since human renin antibodies have cross-reactivity with mouse renin. Plasma renin concentrations (Figure 5E) and systolic blood pressure (Figure 5F) were not different among groups. Western diet induced hypercholesterolemia in all mice. Plasma total cholesterol concentrations (Figure 5G) were not different among groups. Presence of human AGT, human renin, or both human AGT and renin in PTCs did not augment atherosclerotic lesions (Figure 5H).

### Population of human AGT in liver and human renin in renal PTCs had no effect on atherosclerosis in male mice

**Figure 6:**
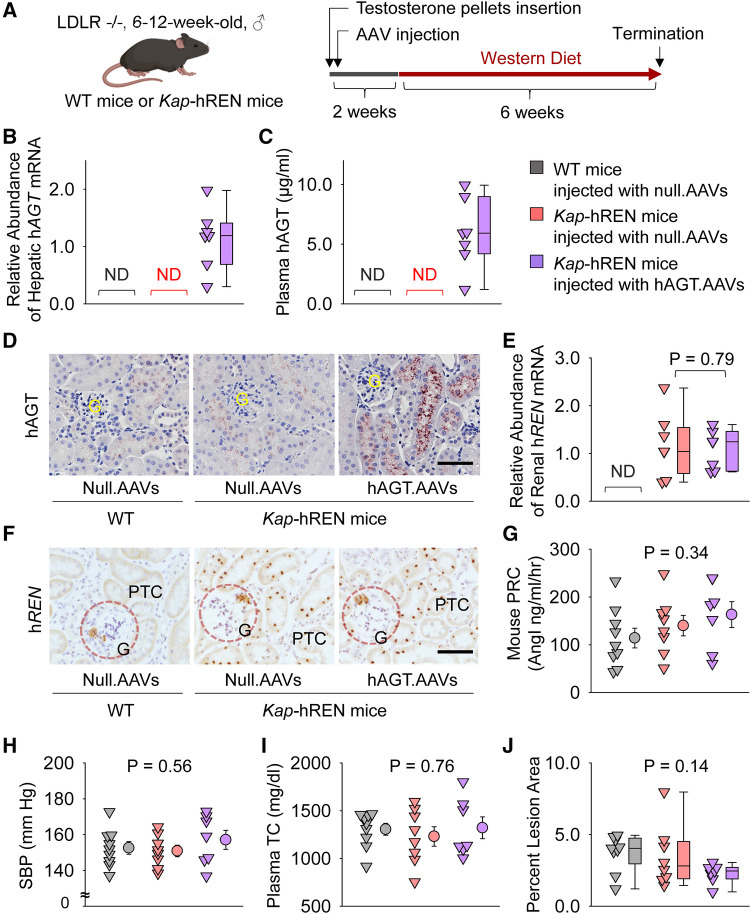
Human AGT in liver and human renin in PTCs had no effect on atherosclerosis in hypercholesterolemic mice. (**A**) Experimental protocol, (**B**) qPCR of renal human AGT (h*AGT*), (**C**) ELISA of plasma human AGT (hAGT), (**D**) immunostaining of human AGT (hAGT) in kidney, (**E**) qPCR of renal human renin (h*REN*), (**F**) RNAscope of human renin (h*REN*) in kidney, (**G**) plasma renin concentrations (PRC), (**H**) systolic blood pressure (SBP), (**I**) plasma total cholesterol concentrations (TC), and (**J**) percent atherosclerotic lesion area. *N* = 7–9/group. Mann–Whitney *U* test was performed to compare renal abundance of h*REN* mRNA between two *Kap-*hREN transgenic mouse groups (**E**) Kruskal–Wallis one-way ANOVA on Ranks test (**G–I**) or one-way ANOVA test (**J**) was performed to compare each parameter among the three groups. ND, not detectable; G, glomerulus; PTC, proximal tubule cells.

Liver AGT is the major contributor to atherosclerosis and liver supplies AGT to renal proximal tubules (13, 32–34). Therefore, we populated human AGT in hepatocytes by intraperitoneally injecting an AAV encoding human AGT (Figure 6A). Since human AGT cannot be cleaved by mouse renin to produce AngI (35), we used *Kap-*hREN transgenic mice to express human renin specifically in PTCs. To activate human renin transgene in PTCs, testosterone pellets were implanted subcutaneously. Two weeks after administration of testosterone and AAVs, mice were fed Western diet for 6 weeks. mRNA of hAGT in liver (Figure 6B) and human AGT protein in plasma (Figure 6C) were only detected in mice infected with hAGT.AAVs. The presence of human AGT in mice infected with hAGT.AAVs was confirmed by immunostaining of human AGT in kidney sections of mice, supporting that human AGT protein was retained in mouse PTCs (Figure 6D). mRNA abundance of human renin was high in *Kap-*hREN mice, but it was not detected in mice without human renin transgene (Figure 6E). The presence of hREN mRNA in PTCs was also evaluated by RNAscope. Since the probe has a cross-reactivity for mouse renin, juxtaglomerular apparatus of both *Kap*-hREN transgenic mice and their wild-type littermate mice showed positive staining; however, positive staining of hREN in PTCs exhibited only in *Kap-*hREN transgenic mice (Figure 6F). Induction of human AGT in liver and human renin in PTCs did not change mouse plasma renin concentrations (Figure 6G) or systolic blood pressure (Figure 6H). All mice were equivalently hypercholesterolemic (Figure 6I) with indistinguishable atherosclerotic lesion size among the 3 groups (Figure 6J).

## Discussion

The present study has three major findings. First, pharmacological inhibition of AT1R by losartan attenuates atherosclerosis in both male and female mice equivalently, although losartan suppresses blood pressure and increases plasma renin concentrations more profoundly in female mice. Second, inducible PTC-specific AT1aR or ACE deletion does not affect systolic blood pressure and atherosclerosis in hypercholesterolemic mice. Third, presence of human AGT and renin in PTCs does not augment atherosclerosis in mice.

AT1 receptor blockers (ARBs) have been used widely to treat high blood pressure and atherosclerosis in both men and women (36, 37). Human and rodent studies implicate that females might be more sensitive to the blood pressure-lowering effects of ARBs. We found that 12.5 mg/kg/d of losartan led to enhanced reductions of blood pressure in female mice, although systolic blood pressure in vehicle group between the two sexes was comparable. It is also interesting that losartan led to higher plasma renin concentrations in female mice than in male mice, implicating stronger negative feedback on AngII and AT1aR interaction in female mice.

Consistent with the effects of ARBs on blood pressure, genetic whole body AT1aR deletion in mice reduced blood pressure. Three studies reported effects of AT1aR deletion in PTCs on blood pressure in mice (38–40). Constitutive deletion of AT1aR on PTCs using *Pepck*-Cre (38) or *Sglt2*-Cre (39) resulted in lower systolic blood pressure, compared to their relative wild type mice; however, blood pressure was higher in mice with PTC-specific deletion of AT1aR than in mice with global AT1aR deletion, indicating that AT1aR in PTCs is not the only contributor to AngII-mediated blood pressure regulation (39). In contrast to blood pressure changes in mice with constitutive PTC-specific AT1aR deletion, testosterone inducible PTC-specific AT1aR deletion in adult mice by *Kap*-Cre did not affect blood pressure (40). This result is consistent with data from using tamoxifen-inducible *Ndrg1*-CreERT2^+/0^ to delete AT1aR on PTCs in adult mice. One possibility is that constitutive vs. inducible deletion of AT1aR may have differential effects on blood pressure regulation. PTCs are composed of two different segments, proximal convoluted tubules (PCTs) and proximal straight tubules (PSTs). X-gal staining illustrated that Cre activity is present in ∼70% of PCTs and all PSTs in ROSA26R^LacZ ^× *Pepck*-Cre^+/0^ mice (41). *Sglt2*-Cre activity was detected in 96% of all PTCs as defined by an anti-megalin antibody (42). *Kap*-Cre is mainly expressed in PTCs of the outer medulla, namely, PSTs (40, 43). We and Endo et al. found that *Ndrg1*-Cre predominantly leads to genetic deletions in PCTs (19). These results indicate that *Pepck*-Cre and *Sglt2*-Cre are more widely expressed in PTCs, compared to *Ndrg1*-Cre and *Kap*-Cre. Therefore, discrepancies of the blood pressure data may also relate to the magnitude of AT1aR deletion in renal proximal tubules.

Hypercholesterolemia leads to the RAS activation and promotes atherosclerosis (3, 44). There is a highly consistent literature that inhibition of ACE (21, 45, 46) or blockade of AT1R (21, 47, 48) have protective effects on hypercholesterolemia-induced atherosclerosis, although most studies only reported male mice. In one study, olmesartan (0.5 or 3 mg/kg/day) was administered to both male and female ApoE (apolipoprotein E) −/− mice fed a high cholesterol diet (49). The dose 0.5 mg/kg/day of olmesartan modestly reduced atherosclerosis in female mice, while 3 mg/kg/day reduced atherosclerosis equivalently in both sexes. In contrast to this study, Zhou et al. reported that female ApoE −/− mice had larger atherosclerotic lesions than male mice, and losartan (5 or 25 mg/kg/day) attenuated atherosclerosis only in female, but not in male, mice (50). We used LDL receptor −/− mice fed Western diet for 12 weeks. Despite the more profound blood pressure reduction in female mice, atherosclerotic lesion area was not different between male and female mice administered losartan. Our findings, in a study that has been performed simultaneously in both male and female mice, are consistent with losartan reducing atherosclerosis independent of blood pressure and sex. However, the mechanisms related to the different regulation of blood pressure and atherosclerosis remain to be defined.

Inhibition of the RAS components attenuates atherosclerosis with reductions in concentrations of renal AngII (12, 14, 21). Despite the associations between renal AngII and atherosclerosis, there has not been a determination whether manipulations in the renal RAS directly impact atherosclerosis. The present study provided direct evidence that AT1aR or ACE deletion on PTCs in adult mice does not affect hypercholesterolemia-induced atherosclerosis. Data generated by RNAscope demonstrated that *Agtr1a* mRNA is present in PTCs, glomeruli, and interstitial cells. AT1aR on podocytes and mesangial cells in glomeruli is important for the homeostasis of the glomerular wall, and interstitial cells communicate with PTCs through a paracrine system (51, 52). As shown by immunostaining, ACE is present predominantly in S3 of PTCs, whereas *Ndrg1*-Cre activation predominantly targets PCTs, but has a modest effect on PSTs. In addition to PTCs, ACE is abundant in endothelial cells of glomeruli and renal vessels. Our previous study has demonstrated that deletion of AT1aR or ACE on endothelial cells does not contribute to atherosclerosis (10, 17). Therefore, we do not predict that the presence of AT1aR or ACE on endothelial cells would affect atherosclerosis in PTC-specific AT1aR or ACE deficient mice. However, the present study cannot rule out that deletion of AT1aR or ACE in the entire kidney contributes to atherosclerosis. Unfortunately, there are no optimal mouse models available to study whether AT1aR or ACE in the whole kidney contributes to hypercholesterolemia-induced atherosclerosis.

Consistent with our findings of genetic deletion, increases of human AGT and renin in PTCs, with either direct increases of human AGT in PTCs (transgenic approach) or via population of human AGT in liver (AAV approach), has no effects on atherosclerosis in hypercholesterolemic mice. Although we have confirmed the high abundance of human AGT protein, we were not able to confirm whether human renin protein was highly abundant in PTCs due to lack of renin antibody specifically targeting human renin. We have also not been able to determine whether human AGT and renin could increase AngII in PTCs. Further studies on whether renal renin-angiotensin components affect atherosclerosis by deletion or increase of either renal AT1aR or AngII production in whole kidney will be considered when appropriate transgenic mice are available.

In summary, losartan leads to different magnitude of responsiveness on plasma renin concentrations and blood pressure between male and female mice, but comparable reductions in atherosclerosis in both sexes. Inducible PTC-specific deletion of AT1aR or ACE or increases of human AGT and renin in adult mice does not affect blood pressure and atherosclerosis in hypercholesterolemic mice.

## Data Availability

The original contributions presented in the study are included in the article/**Supplementary Material**, further inquiries can be directed to the corresponding authors.
