# Supplementary material for: Manipulation of components of the renin angiotensin system in renal proximal tubules fails to alter atherosclerosis in hypercholesterolemic mice
Source: Front Cardiovasc Med. 2023 Aug 16;10:1250234. doi: 10.3389/fcvm.2023.1250234 (PMC10466789; doi:10.3389/fcvm.2023.1250234)
Supplement: Supplementary file 1 [file Datasheet1.pdf]

SUPPLEMENTAL MATERIALS

Manipulation of Components of the Renin Angiotensin System in Renal Proximal Tubules Fails to Alter Atherosclerosis in Hypercholesterolemic Mice

Masayoshi Kukida,[1*] Naofumi Amioka,[1*] Dien Ye,[1] Hui Chen,[1] Jessica J. Moorleghen,[1] Ching-Ling Liang,[1] Deborah A. Howatt,[1] Yuriko Katsumata,[2,3] Motoko Yanagita,[4,5] Hisashi Sawada,[1,6,7] Alan Daugherty,[1,6,7] Hong S. Lu[1,6,7]

[1] Saha Cardiovascular Research Center,
[2] Sanders-Brown Center on Aging,
[3] Department of Biostatistics,
[6] Saha Aortic Center,
[7] Department of Physiology, University of Kentucky, KY, USA

[4] Department of Nephrology, Kyoto University Graduate School of Medicine, Kyoto, Japan
[5] Institute for the Advanced Study of Human Biology (WPI-ASHBi), Kyoto University, Kyoto, Japan

*These authors are joint first authors

Short title: PTC-specific RAS in Atherosclerosis

Corresponding Authors:

Alan Daugherty: Alan.Daugherty@uky.edu

Hong S. Lu: Hong.Lu@uky.edu

MAJOR RESOURCES TABLES

Animals (in vivo studies) – Mice

Figure 1

Genotypes	Sex	Vendor or Source	Strain #	Persistent ID/URL
LDL receptor -/-	Male	The Jackson Laboratory	002207	https://www.jax.org/strain/002207
LDL receptor -/-	Female	The Jackson Laboratory	002207	

Figure 2B

Genotypes	Sex	Vendor or Source
ROSA26R^{LacZ} *Ndrg1*-CreERT2$^{0/0}$	Male	In house breeding
ROSA26R^{LacZ} *Ndrg1*-CreERT2$^{+/0}$	Male	In house breeding

Figure 2C

Genotypes	Sex	Vendor or Source
ROSA26R$^{mT/mG}$ *Ndrg1*-CreERT2$^{0/0}$	Male	In house breeding
ROSA26R$^{mT/mG}$ *Ndrg1*-CreERT2$^{+/0}$	Male	In house breeding

Figure 3

Genotypes	Sex	Vendor or Source
Agtr1a f/f *Ndrg1*-CreERT2$^{0/0}$ LDL receptor -/-	Male and Female	In house breeding
Agtr1a f/f *Ndrg1*-CreERT2$^{+/0}$ LDL receptor -/-	Male and Female	In house breeding

Figure 4

Genotypes	Sex	Vendor or Source
Ace f/f *Ndrg1*-CreERT2$^{0/0}$ LDL receptor -/-	Male and Female	In house breeding
Ace f/f *Ndrg1*-CreERT2$^{+/0}$ LDL receptor -/-	Male and Female	In house breeding

Figure 5

Genotypes	Sex	Vendor or Source
LDL receptor -/- (wild-type control)	Male	In house breeding
Kap-hAGT x LDL receptor -/-	Male	In house breeding
Kap-hREN x LDL receptor -/-	Male	In house breeding
Kap-hAGT x *Kap*-hREN x LDL receptor -/-	Male	In house breeding

Figure 6

Genotypes	Sex	Vendor or Source
LDL receptor -/- (wild-type control)	Male	In house breeding
Kap-hREN x LDL receptor -/-	Male	In house breeding

Mouse Breeding Pairs

Figure 2B

Genotypes	Sex	Vendor or Source
Ndrg1-CreERT2$^{+/0}$	Male	In house breeding
ROSA26R^{LacZ}	Female	The Jackson Laboratory (Strain # 003474)

Figure 2C

Genotypes	Sex	Vendor or Source
Ndrg1-CreERT2$^{+/0}$	Male	In house breeding
ROSA26R$^{mT/mG}$	Female	The Jackson Laboratory (Strain # 007676)

Figure 3

Genotypes	Sex	Vendor or Source
Agtr1a f/f *Ndrg1*-CreERT2$^{+/0}$ LDL receptor -/-	Male	In house breeding
Agtr1a f/f *Ndrg1*-CreERT2$^{0/0}$ LDL receptor -/-	Female	In house breeding

Figure 4

Genotypes	Sex	Vendor or Source
Ace f/f *Ndrg1*-CreERT2$^{+/0}$ LDL receptor -/-	Male	In house breeding
Ace f/f *Ndrg1*-CreERT2$^{0/0}$ LDL receptor -/-	Female	In house breeding

Figure 5

Genotypes	Sex	Vendor or Source
Breeding Strategy 1		
Kap-hAGT x LDL receptor -/-	Male	In house breeding
Kap-hREN x LDL receptor -/-	Female	In house breeding
Breeding Strategy 2		
Kap-hREN x LDL receptor -/-	Male	In house breeding
Kap-hAGT x LDL receptor -/-	Female	In house breeding

Figure 6

Genotypes	Sex	Vendor or Source
Breeding Strategy 1		
LDL receptor -/-	Male	In house breeding
Kap-hREN x LDL receptor -/-	Female	In house breeding
Breeding Strategy 2		
Kap-hREN x LDL receptor -/-	Male	In house breeding
LDL receptor -/-	Female	In house breeding

Primer Sequences for Genotyping

Gene	Vendor or Source	Primer Sequence (5'-3')
hAGT	Integrated DNA Technologies	Forward: TGG TGC TAG TCG CTG CAA AAC TTG ACA CCG<br>Reverse: CAG GGA GCA GCC AGT CTT CCA TCC TGT CAC
hREN	Integrated DNA Technologies	Forward: TGA CAC TGG TTC GTC CAA TG<br>Reverse: ATA GCG GAG GGT GAG TTC TG
Cre	Integrated DNA Technologies	Forward: ACC TGA AGA TGT TCG CGA TT<br>Reverse: CGG CAT CAA CGT TTT CTT TT
IL-2	Integrated DNA Technologies	Forward: CTA GGC CAC AGA ATT GAA AGA TCT<br>Reverse: GTA GGT GGA AAT TCT AGC ATC ATC C

Primer Information (TaqMan Probes) for Quantitative Polymerase Chain Reaction (qPCR)

Gene	Vendor or Source	Catalog #
Ace	Thermo Fisher Scientific	Mm00802048_m1
Actb	Thermo Fisher Scientific	Mm01205647_g1
Gapdh	Thermo Fisher Scientific	Mm99999915_g1
Ppia	Thermo Fisher Scientific	Mm02342429_g1

Primer Information for qPCR using SYBR Green Method

Gene	Vendor or Source	Primer Sequence (5'-3')
Agtr1a	Integrated DNA Technologies	Forward: GAC CAA CTC AAC CCA GAA AAG C<br>Reverse: ATC ACC ACC AAG CTG TTT CC
hAGT	Integrated DNA Technologies	Forward: GAA CTG GAT GTT GCT GCT GA<br>Reverse: GGA GAA GCC CTT CAT CTT CC
hREN	Integrated DNA Technologies	Forward: CAA GGG GTG CTA AAA GAG GA<br>Reverse: CTG CCA GAC ACC AGT CTT GA
Actb	Integrated DNA Technologies	Forward: GCC TTC CTT CTT GGG TAT GG<br>Reverse: GCA CTG TGT TGG CAT AGA GG
Gapdh	Integrated DNA Technologies	Forward: CAA CTC CCA CTC TTC CAC CT<br>Reverse: CTT GCT CAG TGT CCT TGC TG
Rplp2	Integrated DNA Technologies	Forward: ATG TCA TCG CTC AGG GTG TT<br>Reverse: CTC CTC GGA CTC CTC CTT CT

Primary Antibodies for Immunostaining

Target antigen	Vendor	Catalog #	Working concentration
Mouse angiotensin-converting enzyme	abcam	ab254222	0.1 μg/ml
Human angiotensinogen	abcam	ab276132	0.1 μg/ml

Probes for RNAscope

Reagents	Vendor or Source	Catalog #
Mouse *Agtr1a*	Advanced Cell Diagnostics	481161
Human *REN*	Advanced Cell Diagnostics	401921

Animal Study Information Following the ARRIVE Essential 10

Figure 1. All mice were LDL receptor -/-

Groups	Sex	Age (weeks)	Number (prior to experiment)	Number (termination)	Littermates (Yes/No)
Vehicle	M	8-10	10	10	Yes
Losartan 12.5 mg/kg/d	M	8-10	10	10	
Vehicle	F	8-10	9	10	
Losartan 12.5 mg/kg/d	F	8-10	10	10	

Figure 3B. All mice were in an LDL receptor -/- background

Groups	Sex	Age for tamoxifen injection (weeks)	Number (prior to experiment)	Number (termination)	Littermates (Yes/No)
Agtr1a f/f *Ndrg1*-CreERT2$^{0/0}$	M	4-6	7	7	Yes
Agtr1a f/f *Ndrg1*-CreERT2$^{+/0}$	M	4-6	4	4	
Agtr1a f/f *Ndrg1*-CreERT2$^{0/0}$	F	4-6	7	7	
Agtr1a f/f *Ndrg1*-CreERT2$^{+/0}$	F	4-6	4	4	

Figure 3D-G. All mice were in an LDL receptor -/- background

Groups	Sex	Age for tamoxifen injection (weeks)	Number (prior to experiment)	Number (termination)	Littermates (Yes/No)
Agtr1a f/f *Ndrg1*-CreERT2$^{0/0}$	M	4-6	13	13	Yes
Agtr1a f/f *Ndrg1*-CreERT2$^{+/0}$	M	4-6	11	11	
Agtr1a f/f *Ndrg1*-CreERT2$^{0/0}$	F	4-6	11	11	
Agtr1a f/f *Ndrg1*-CreERT2$^{+/0}$	F	4-6	11	11	

Figure 4B, D-F. All mice were in an LDL receptor -/- background

Groups	Sex	Age for tamoxifen injection (weeks)	Number (prior to experiment)	Number (termination)	Littermates (Yes/No)
Ace f/f *Ndrg1*-CreERT2$^{0/0}$	M	4-6	10	10	Yes
Ace f/f *Ndrg1*-CreERT2$^{+/0}$	M	4-6	12	12	
Ace f/f *Ndrg1*-CreERT2$^{0/0}$	F	4-6	13	13	
Ace f/f *Ndrg1*-CreERT2$^{+/0}$	F	4-6	7	7	

Figure 5B-C and E-H. All mice were in an LDL receptor -/- background

Groups	Sex	Age (weeks)	Number (prior to experiment)	Number (termination)	Littermates (Yes/No)
Wild-type	M	7-9	6	6	Yes
Kap-hAGT	M	7-9	4	4	
Kap-hREN	M	7-9	3	3	
Kap-hAGT x *Kap*-hREN	M	7-9	4	4	

Figure 6B-C, E, G-J. All mice were in an LDL receptor -/- background

Groups	Sex	Age (weeks)	Number (prior to experiment)	Number (termination)	Littermates (Yes/No)
Wild-type injected with null.AAVs	M	6-12	9	9	Yes
Kap-hREN injected with null.AAVs	M	6-12	8	8	
Kap-hREN mice injected with hAGT.AAVs	M	6-12	7	7	

Item	Application
Ethics	Approved by the University of Kentucky IACUC (2018-2968).
Sex	Most studies included both male and female mice. In the experiments using testosterone pellets (Figures 5 and 6), only male mice were studied due to the frequent occurrence of uterine prolapse in female mice following testosterone pellet implantation, which were required to be euthanatized prior to reaching the endpoint based on the IACUC regulation.
Inclusion criteria	Based on sex, age, body weight, and overt health appearance in each experiment.
Exclusion criteria	Based on sex, age, and body weight, Medical cases reported by a veterinarian
Sample size	Described in each figure legend.
Sample size calculation	None
Primary endpoint	Atherosclerosis in aortas
Randomization	Study mice were numbered and grouped randomly based on their genotypes
Blinding	Quantification of atherosclerosis was verified by an independent investigator blinded to the study group information.
Statistical analysis	SigmaPlot version 14.5 or 15.0 (SYSTAT Software Inc., CA), or R Statistical Software (v4.1.1; R Core Team 2021)
Statistical method	Described in each figure legend
Data availability	All numerical data used for figures are available in Supplemental Excel File.
